# The Role of Compounds Derived from Natural Supplement as Anticancer Agents in Renal Cell Carcinoma: A Review

**DOI:** 10.3390/ijms19010107

**Published:** 2017-12-31

**Authors:** Inamul Haque, Arvind Subramanian, Chao H. Huang, Andrew K. Godwin, Peter J. Van Veldhuizen, Snigdha Banerjee, Sushanta K. Banerjee

**Affiliations:** 1 Department of Pathology and Laboratory Medicine, University of Kansas Medical Center, Kansas City, KS 66160, USA; arvind.subramanian@hotmail.com (A.S.); agodwin@kumc.edu (A.K.G.); sbanerjee@kumc.edu (S.B.); 2 Medical Oncology, Department of Internal Medicine, University of Kansas Medical Center, Kansas City, KS 66160, USA; chao.huang@va.gov; 3 Cancer Research Unit, Kansas City Veterans Affairs Medical Center, Kansas City, MO 64128, USA; 4 Division of Clinical Oncology, Kansas City Veterans Affairs Medical Center, Kansas City, MO 64128, USA; peter.vanveldhuizen@va.gov; 5 University of Kansas Cancer Center, Kansas City, KS 66160, USA; 6 Department of Anatomy and Cell Biology, University of Kansas Medical Center, Kansas City, KS 66160, USA

**Keywords:** renal cell carcinoma, natural products, Epigallocatechin-3-gallate (EGCG), Englerin A, Quercetin, miRNA

## Abstract

Renal Cell Carcinoma (RCC) is the most prominent kidney cancer derived from renal tubules and accounts for roughly 85% of all malignant kidney cancer. Every year, over 60,000 new cases are registered, and about 14,000 people die from RCC. The incidence of this has been increasing significantly in the U.S. and other countries. An increased understanding of molecular biology and the genomics of RCC has uncovered several signaling pathways involved in the progression of this cancer. Significant advances in the treatment of RCC have been reported from agents approved by the Food and Drug Administration (FDA) that target these pathways. These agents have become drugs of choice because they demonstrate clinical benefit and increased survival in patients with metastatic disease. However, the patients eventually relapse and develop resistance to these drugs. To improve outcomes and seek approaches for producing long-term durable remission, the search for more effective therapies and preventative strategies are warranted. Treatment of RCC using natural products is one of these strategies to reduce the incidence. However, recent studies have focused on these chemoprevention agents as anti-cancer therapies given they can inhibit tumor cell grow and lack the severe side effects common to synthetic compounds. This review elaborates on the current understanding of natural products and their mechanisms of action as anti-cancer agents. The present review will provide information for possible use of these products alone or in combination with chemotherapy for the prevention and treatment of RCC.

## 1. Introduction

Renal Cell Carcinoma (RCC) is a disease found in the lining of the kidney tubules [1]. It is the most prominent kidney cancer in adults, accounts for roughly 85% of all malignant kidney cancer, and can result in a number of symptoms, including weight loss, fever, hypertension, hypercalcemia, night sweats, and malaise [2,3]. Although RCC is quite rare, it is still among the top ten cancers, often affecting people over 45 years old [4]. More specifically, this cancer affects men more than women, with the average age of diagnosis being around 60 years [5,6]. Its incidence rates have been gradually increasing by 2–4% every year over the past few decades [7]. The most current cancer data estimate that nearly 64,000 new cases of renal cancer will be diagnosed in the United States, while about 14,400 people will die from complications associated with renal cancer in 2017 [8]. The five-year survival rate for the patients with this disease is approximately 85% if detected and treated early, while it is only 10% when it is detected at later stages [9]. An increased understanding of molecular biology and genomics of RCC have identified several signaling pathways involved in the progress of this disease [10]. Significant advances in the treatment of RCC have been derived from agents approved by the FDA that target several pathways. These include inhibitors of mammalian target of rapamycin (mTOR) (e.g., everolimus and temsirolimus) and the tyrosine kinase inhibitors (TKIs) (e.g., sorafenib, sunitinib, pazopanib and axitinib). Everolimus and temsirolimus block the activation of AKT, hypoxia inducible factor α (HIFα) and p70S6 kinase by targeting mTOR complex 1 and 2 (mTORC1 and mTORC2), and in turn inhibit cell growth and survival. Sorafenib, sunitinib, pazopanib and axitinib target multiple pro-angiogenic growth factors such as vascular endothelial growth factor (VEGF) and platelet derived growth factor (PDGF), and their receptors VEGFR and PDGFR. These agents were approved by the FDA because they demonstrate clinical activity and increased survival in patients with metastatic disease [11,12,13]. These drugs showed clinical benefit without adversely impacting overall quality of life and had a positive impact on specific symptoms, e.g., cough, fevers, shortness of breath, ability to enjoy life, and worry that the condition will get worse in patients with advanced RCC. However, patients eventually relapse and develop resistance to these drugs [12,14,15]. To reduce the death associated with RCC, it would be important to improve methods for detection, prevention, and treatment. In this review, we will evaluate natural products traditionally studied in chemoprevention, i.e., the use of chemicals, bioactive plant compounds or dietary components to block, inhibit or reverse the development of cancer in normal or preneoplastic tissue, as therapies for the treatment of RCC.

**Figure 1:**
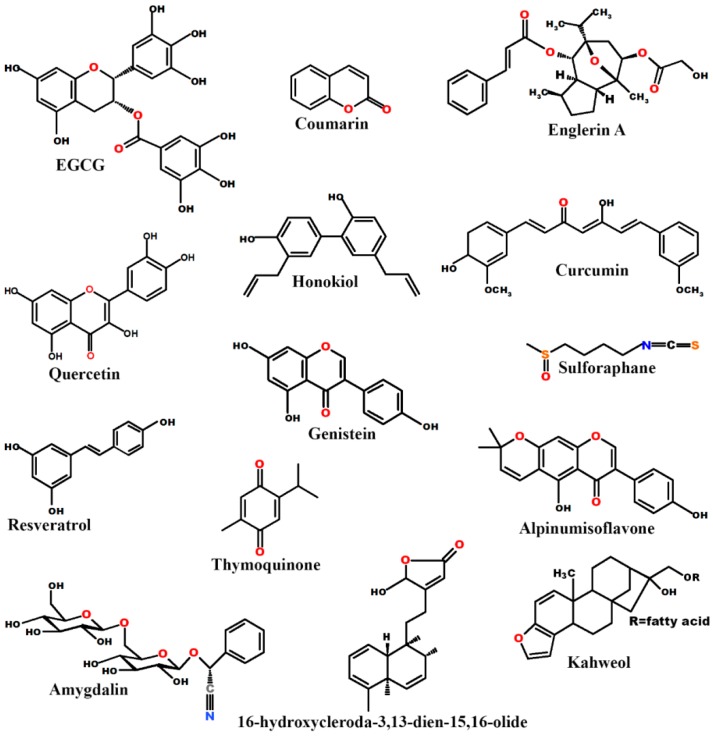
Chemical structures of some commonly studied natural products.

Previous studies have found that many compounds originated from natural products could be used as both preventive and therapeutic agents. In combination with chemotherapy or alone, they have been shown to enhance the efficacy and tolerance of the chemotherapeutic agents in various cancers [16,17,18,19,20,21,22]. This review article will elaborate on our current understanding of the effectiveness of naturally occurring anti-cancer agents in the treatment of RCC. We summarize studies on the effects of Epigallocatechin-3-gallate (EGCG), Englerin A, Quercetin, coumarins, curcumin, and other natural products against RCC. The structures of these natural products are shown in Figure 1.

## 2. Natural Products and Renal Cell Carcinoma

Natural products have been used for thousands of years for their medicinal properties [23], yet researchers have only recently started investigating the role they play at a molecular level. These natural products are significant because their use in ancient history can be validated and applied to modern therapy with evidenced conclusions. Indeed, nearly every major ancient civilization has used some form of natural products as traditional medicines, remedies, potions and oils with many of these bioactive natural products still being unidentified [24]. The earliest records of the use of natural products for medicinal purposes can be traced to 2600 B.C., when documented oils from *Cupressus sempervirens* (cypress) and *Commiphora* species (myrrh) were used to treat illnesses [25].

Recent medical history has placed natural products on the back-burner, nearly always preferring human-made drugs derived from the study of molecular biology and combinatorial chemistry [26]. However, these drugs can often be extremely expensive [27]. Furthermore, they usually have intolerable side effects that make them prohibitive to treat human diseases, including having the opposite of the intended effect [23]. In general, herbal or natural treatments have few to no side effects while producing very favorable results in tumor treatment [23]. However, the therapeutic activities of the compounds within these products have not been studied extensively in RCC. It is thus prudent to investigate the pathways affected by compounds in these natural products.

### 2.1. Epigallocatechin-3-Gallate

A significant distinction between normal healthy cells and tumor cells is that the latter often circumvent the apoptosis process, allowing uncontrolled proliferation. Thus, inducing apoptosis would be an effective means of treatment. In RCC, the expression of tissue factor pathway inhibitor-2 (TFPI-2) is inversely related to the aggressiveness of these cells [28]. Therefore, higher concentrations of TFPI-2 would decrease the malignancy of these cells and most likely induce apoptosis. Epigallocatechin-3-gallate (EGCG), an active and major constituent of green tea (*Camellia sinensis*), displays anti-tumor properties in several cancers, including RCC [29,30,31,32,33,34,35] and inhibits tumor growth and invasiveness in RCC by upregulating expression of TFPI-2 through inhibition of DNA methyltransferase (DNMT) activity [28].

A recent paper indicates that EGCG may play a preventive role in the development of RCC [36]. This study evaluated the effect of tumor necrosis factor-related apoptosis-inducing ligand (TRAIL), EGCG, and a combination of both on a TRAIL-resistant RCC cell line, 786-O. The data demonstrate that EGCG alone provided a significant reduction in cell viability, but co-treatment with TRAIL provided a marked reduction in cell viability greater than that of EGCG or TRAIL alone by downregulating c-FLIP, MCl-1, and BCl-2.

Another study reported that EGCG induces apoptosis, inhibiting the proliferation and migratory potential of RCC cell lines by downregulating the expression of matrix metalloproteinase-2 (MMP-2) and matrix metalloproteinase-9 (MMP-9) [37]. However, this study did not determine how the expression levels and activity of these metalloproteinases are regulated by EGCG.

It is clear through multiple, independent experiments that EGCG has proven an extremely viable treatment in vitro. A few methods to utilize EGCG arise from the data previously presented. One example was an extensive epidemiological study which reported an inverse correlation between green tea consumption and overall RCC tumor burden [38]. Another approach might use EGCG in supplement with TKI or mTOR inhibitors to see if the combination particularly sensitizes the tumor cells as compared to TKI or mTOR inhibitor alone [39,40,41,42]. A study by Sato et al., suggests that the restoration of connexin 32 (*Cx32*) gene, a tumor suppressor, by EGCG pretreatment enhanced the chemical sensitivity of vinblastine via the inactivation of Src and the activation of the c-Jun NH2-terminal kinase (JNK) in RCC cells [43]. Overall, these studies suggest that EGCG could be used as both a preventative and therapeutic approach for renal cell carcinoma.

### 2.2. Englerin A

Englerin A is a natural product derived from the root and stem bark of *Phyllanthus engleri*, an indigenous African plant. It was identified to preferentially inhibit the cell growth and viability of RCC through a drug screen of the NCI 60 (National Cancer Institute 60) cell line panel [44]. This natural product is a guaiane sesquiterpene with a tricyclic structure that has a standardized procedure for synthesis in a laboratory [45].

There are multiple proposed mechanisms for the RCC growth inhibition by Englerin A which have been summarized in detail in a comprehensive review by Beutler and coworkers [46]. One such proposal comes from Ramos’s group, which suggests that growth of RCC cell lines can be inhibited by Englerin A through necrotic cell death rather than apoptosis [47]. The authors report that apoptotic bodies, typical in apoptotic cell death, were not present after Englerin A treatment. Calcium ions have been associated with necrotic cell death [48]. Ramos’s group tested calcium ion content in various renal cell carcinoma cell lines and found that SF-295 cells showed little relative change in ion content while A-498 cells showed a four-fold increase in concentration [47]. Although this study indicates that apoptotic bodies were not present, Williams et al., suggest otherwise [49]. Not only necrosis was observed, but apoptosis and autophagy were also noticed after 24 h of treatment in A498 cells [49]. In addition, their results also suggest that Englerin A-induced inhibition of RCC growth was due to cell cycle arrest by blocking G2/M transition and suppression of AKT and ERK activity.

Englerin A triggers the activity of the enzyme protein kinase C θ (PKCθ), which has been shown in vitro to phosphorylate and activate heat shock factor 1 (HSF1), resulting in insulin resistance and glucose deprivation of 786-O cells [50]. However, PKCθ is not expressed in A498 cells which are the most sensitive to Englerin A [51]. This finding led researchers to investigate other possible targets. Reports from two independent groups suggested that the transient receptor potential cation channel, subfamily C, member 4/5 (TRPC4/5) are characteristic to Englerin A sensitivity, and thus indicate these may be targets of Englerin A [51,52]. The authors claim that Englerin A induces cell death by the elevated Ca^2+^ influx and membrane depolarization, which occurred much more frequently in cells that expressed high levels of TRPC4 on their surface [52]. However, a recent finding contradicts these results and suggests that Enlerin A cytotoxicity is mediated by the influx of Na^+^ through TRPC4/TRPC1 channels [53].

A hallmark of metastasis in malignancies including renal cancer is the epithelial-mesenchymal transition (EMT), followed by invasion [54,55]. In our group, we sought to examine the effects of Englerin A in preventing the migration and invasion of RCC cell lines as well as to investigate whether Englerin A may inhibit the molecular changes associated with EMT induced by transforming growth factor-β1 (TGF-β1) [56]. We also aimed to see whether Englerin A suppresses cancer stem cell markers and spheroid formation. Our results show that Englerin A inhibits molecular changes associated with TGF-β1-induced EMT by upregulating the epithelial markers and downregulating the mesenchymal/stem cell markers [56]. We also found that Englerin A inhibits TGF-β1-induced angiogenesis. This study indicated that Englerin A might serve as a potential candidate for the treatment of renal cancer metastasis.

In a recent study, Batova and colleagues proposed a different mechanism for RCC cell death by Englerin A [57]. They demonstrated that Englerin A alters lipid metabolism, induces (endoplasmic reticulum) ER stress, and in turn generates an excess of ceramides, which are lethal to RCC cells. Furthermore, Englerin A induces an acute inflammatory response.

Little work has been conducted regarding in vivo models, and those that have been conducted on mouse models indicate that the levels of Englerin A required for anti-tumor activity may be lethal [52,58]. If the results of this in vivo model accurately reflect the natural product’s effects, this would be a major impediment for its use in cancer treatment. However, the compound itself is certainly worth investigating. It would be extremely effective in treatment if a non-lethal derivative of Englerin A was found and implemented. Further, there is still an ongoing debate as to which mechanisms Englerin A uses to elicit anti-tumor effects. If it is found that Englerin A uses many pathways for tumor suppression, its use could be used extended to treat other tumors that use these pathways.

### 2.3. Quercetin

Quercetin (3,3’,4’,5,7-pentahydroxyflavone) is part of a class of pigments called flavonoids that is found in many food items, such as tea, onions, grapes, and apples [59]. Quercetin itself has been shown to exhibit a chemopreventive role in several cancers including liver, lung, prostate cancers, breast and renal cancer [60,61,62,63,64,65]. This natural product has proven very effective when used in combination with other compounds [66,67]. Quercetin has a therapeutic effect when used with hyperoside in 786-0 renal cancer cells [66]. The mechanism for this activity involves downregulation of miRNA-27a, a mechanism that we have not yet explored in this article. Most natural products we have considered trigger apoptosis or necrosis using other pathways. Meanwhile, the reduction in miRNA-27a combined with an increase in ZBTB10 (the zinc finger and BTB domain-containing protein 10) triggers a decrease in specificity protein (SP) transcription factors [66]. These transcription factors are highly expressed in cancer cells, and their reduction shows the therapeutic potential of quercetin.

Methylation by catechol-*O*-methyltransferase (COMT) enzyme significantly decreased the chemopreventive activity of EGCG in several cancers [68,69,70]. Quercetin has been reported to increase the activity of EGCG in terms of bioavailability in animal models by inhibiting COMT activity [65].

Snail is a zinc-finger transcription factor and plays a key role in EMT, migration and metastasis [71,72]. Its silencing by short hairpin RNA (shRNA) inhibits cellular proliferation, cell cycle progression, cancer cell migration and promoted apoptosis in Caki-2 cell lines [67]. Quercetin together with snail silencing provides even strong suppressive effects toward these cells. Quercetin has significant therapeutic potential that can be honed through research and more thorough investigation.

Isoquercetin, which is hydrolyzed in vivo to quercetin is currently being assessed in combination with sunitinib (clinicaltrials.gov: NCT02446795). In this ongoing clinical trial, the investigators hypothesized that isoquercetin is able to reduce sunitinib-induced fatigue which is being reported in 51–63% of advanced RCC patients.

### 2.4. Coumarin

Coumarin (1,2-benzopyrone) belongs to a benzopyrone family of compounds found in different parts of plants, having the highest concentration in fruits, followed by roots, seeds, and leaves. It can also be synthesized in the laboratory [73,74]. Researchers continue to show a strong interest in coumarin and its derivatives because of their diverse pharmacological and biological properties such as anti-thrombic, scavenging of reactive oxygen species, anti-mutagenic, anti-bacterial, cycloxygenase inhibition as well as an anti-tumorigenic effect [75].

Multiple studies have demonstrated that coumarins possess cytostatic and cytotoxic properties, inhibiting growth in several human cancer cell lines in vitro. In some clinical trials, they have shown anti-proliferative activities against several cancers including RCC [74,76,77,78,79,80,81]. Keeping in mind the anti-neoplastic action of coumarins [82], Myers et al., found that coumarin in vitro inhibited the proliferation of RCC cells [83]. Coumarins isolated from *Calophyllum dispar* has been used as traditional medicine to treat RCC [84]. Reduction in metastatic development among patients with RCC was noted when coumarin was given orally [82]. A derivative consisting of 1,2,4-triazolin-3-one attached to 4-methylcoumarin was found to have encouraging activity against RCC cell line [85]. A recent derivative, coufin, a novel indolylcoumarin, showed potent anticancer activity both in 2D (monolayer culture) and 3D (tumor spheroid culture) by inhibiting microtubule formation and blocking the cell cycle at G2/M [86].

Since coumarin has low toxicity, there is a scientific rationale for using coumarin with other compounds in an attempt to increase their efficacies [87]. A pilot study by Marshall et al., reported a beneficial effect of coumarin and cimetidine in RCC patients [77]. In a clinical study, patients with metastatic RCC were given interferon-α (IFN-α) plus coumarin and cimetidine, or IFN-α-monotherapy [88]. This study claims that using coumarin plus cimetidine to IFN-α did not increase response rates or survival of the patients. Further studies need to be performed to resolve the potential therapeutic value of coumarins in combination with other agents.

### 2.5. Curcumin

Curcumin (1,7-bis(4-hydroxy-3-methoxyphenyl)-1E,6E-heptadiene-3,5-dione or diferuloyl methane), a natural polyphenolic phytochemical isolated from dried rhizomes of turmeric plant (*Curcuma longa*) has been used for centuries as traditional Indian and Chinese medicine for the treatment of a variety of diseases [89,90,91]. Across a variety of studies, curcumin has shown numerous pharmacological activities, including anti-inflammatory [92,93,94], antiviral [95,96], anti-oxidant [94], wound healing [97], hepatoprotective [98], and anti-microbial effects [99,100]. Moreover, curcumin has been used as a chemopreventive agent and an anti-cancer therapy in several human carcinomas, including colorectal [101], melanoma [102], lymphoma [103], breast [104,105], thyroid [106], head and neck [107], prostate [108], pancreatic [109,110], ovarian [111] and RCC [91,112,113,114,115,116,117,118].

Curcumin has been reported to efficiently induce apoptosis in vitro in various human cancer cell lines [18,119,120,121]. The mechanism(s) by which curcumin can induce apoptosis in RCC cells to remain poorly understood. Initial reports by Kim et al., suggest that curcumin induces apoptosis in Caki cells by activating caspase 3 and releasing mitochondrial cytochrome C [122]. Woo et al., also suggested that curcumin induced apoptosis through the dephosphorylation of AKT, down-regulation of BCL-2, BCL-XL and inhibitor of apoptosis protein (IAP) proteins, activation of caspase 3 and release of cytochrome C [114]. Zhang and groups demonstrated that curcumin significantly inhibits proliferation of RRC-949 cell lines and induces cell apoptosis, possibly via regulation of BCL-2 and BAX, and initiates cell cycle arrest in G2/M phase [116]. Curcumin exposure induces apoptosis through cell cycle arrest in G1-phase and increases the volume of human kidney cells by modulating chloride ion channel [91].

Moreover, curcumin has been proven to increase the efficacy of chemotherapeutic drugs. Since PI3K/AKT and mechanistic target of rapamycin (mTOR) signaling are hyper-activated in RCC, inhibition of these pathways is warranted for RCC treatment [123]. Although NVP-BEZ235 inhibits PI3K/AKT and mTOR pathways, it was not sufficient to induce apoptosis in RCC cell lines [113]. Curcumin significantly induces apoptosis in NVPBEZ235-treated cells through p53-dependent downregulation of MCL-1 and BCL-2 protein expression [113]. However, the exact mechanism continues remains unclear.

Yes-associated protein (YAP), the effector of the Hippo signaling pathway, is reported either as an oncogene or a tumor suppressor and plays contradictory roles in the development of cancer [124]. Reports from Bai et al., indicate that YAP functions as a tumor suppressor that enhances chemosensitivity via apoptosis by modulating p53 during chemotherapy [124]. Short hairpin RNA-mediated knockdown of YAP significantly inhibited cell proliferation, migration, and colony formation efficiency of RCC cells in soft agar and led to significantly reduced tumor growth in mice by activating p53 signaling and inhibiting mitogen-activated protein kinase (MAPK) signaling [125]. However, Xu et al., reported that combined treatment with curcumin and temsirolimus in Caki-1 and OS-RC-2 RCC cell lines markedly upregulates YAP, which binds to p53 promoter, enhances p53 expression and finally induces apoptosis by activation of cleaved poly ADP-ribose polymerase (PARP) and caspase 3, and downregulation of BCL-2 protein expression [115]. Curcumin sensitizes human renal cancer cells to tumor necrosis factor-related apoptosis-inducing ligand (TRAIL)-induced apoptosis by upregulating death receptor 5 (DR5) expression and generating reactive oxygen species (ROS) [126]. The results from these studies suggest that using curcumin is a potentially novel and efficient strategy to enhance the effectiveness of targeted drugs in human RCC.

Although curcumin has been successfully proven to be very effective in vitro in diminishing cancer cell proliferation, migration, and invasion, it exhibits lesser effects in vivo due to poor bioavailability, poor absorption, rapid metabolism in liver cells and intestinal wall. Several strategies such as novel drug delivery systems, blocking of metabolic pathways, and synthesis of curcumin analogs have been explored in attempting to improve the bioavailability and gain in its metabolic stability [112,127,128,129,130,131,132]. We anticipate that use of curcumin or its analogs in clinics for the prevention and/or treatment of RCC and other cancers.

### 2.6. Resveratrol

Resveratrol (trans 3,4′,5-trihydroxystilbene) is a naturally occurring polyphenolic compound found in grapes and 72 additional plant species and is relatively abundant in red wines [133]. It has been reported to induce apoptosis, inhibit tumor growth, and suppress angiogenesis and metastasis in various malignancies including RCC [134]. The results from microarray gene expression profiling revealed that resveratrol modulates the genes related to the inhibition of cell growth and induction of apoptosis [133]. It has been indicated that resveratrol significantly inhibits the RRC cell proliferation and exerts an antitumor effect by concomitant inhibition of the expression of VEGF, a vital feature of RCC microenvironment [135]. In a recent study, Kim et al. [134] demonstrated the pro-apoptotic and anti-invasive role of resveratrol in RCC, and their results suggest that it suppresses the activation of signal transducers and activators of transcription 3/5 (STAT3/5) proteins, which are aberrantly activated in RCC [136]. Furthermore, resveratrol induced S-phase arrest and apoptosis, decreased mitochondrial membrane potential, and suppressed colony formation in RCC. They also found that resveratrol shows caspase 3-mediated apoptosis, and blockage of metastasis by downregulating the expression of BCL-2, BCL-XL, IAP1/2, survivin, COX-2, MMP2 and VEGF.

Besides, resveratrol increases sorafenib induced inhibitory effect on phosphorylation of STAT3/5, apoptosis, and in turn results in downregulation of various oncogenic gene products. In addition to its antitumor action, resveratrol can exhibit antitumor immune response. It boosts antitumor immunity in mice by efficiently suppressing regulatory T cells (Tregs), inhibiting TGF-β level, and increasing IFN-γ expressing CD8^+^ T cells [137]. In agreement to these results, Chen et al., reported that resveratrol reduces Tregs cells, stimulates cytotoxic CD8^+^ T cells, increases IFN-γ and reducing the level of interleukin-6 (IL-6) and IL-10 [138]. Moreover, they also revealed that resveratrol suppresses tumor growth by significantly inhibiting abnormal angiogenesis by downregulating VEGF level. Taken together, resveratrol may, therefore, be an effective antitumor therapy drug and improve outcomes for RCC patients.

### 2.7. Other Natural Products

There are additional natural bioactive products which possess anticancer activities against RCC. Honokiol ((3′,5-di-(2-propenyl)-1,1′-biphenyl-2,2′-diol) is a biologically active biphenolic compound isolated from *Magnolia spp*. bark, which has been extensively used in traditional Chinese medicine and shown to exhibit an anticancer effect in various cancer [139,140,141,142,143,144]. However, very limited literature is available which deciphers the anticancer role of honokiol in RCC. Honokiol suppresses cell proliferation and migration of highly metastatic RCC cell line, 786-0 through activation of RhoA/ROCK/MLC signaling [145]. Li et al., demonstrated that honokiol inhibits metastasis through reversing EMT and suppressing cancer stem cell (CSC) properties via modulating miR-141/ZEB2 axis [146]. Another group also shows that honokiol suppresses the invasion and metastasis by upregulating the expression of metastasis suppressor genes like *KISS-1*, *TIMP4*, *KISS-1R* and *TP53*, and concomitant downregulating *CXCL12*, *CCL7*, *IL-18*, and *MMP7* expression in RCC cells [145]. These studies suggest that honokiol may be a suitable therapeutic approach for RCC treatment.

Genistein (4′,5,7-trihydroxyflavone or 5,7-dihydroxy-3-(4-hydroxyphenyl)-4H-chromen-4-one) is one of the principal isoflavones found in soybeans. Many studies have shown that genistein inhibits several cancers by modulating different signaling pathways involved in cell cycle progression, apoptosis, invasion, angiogenesis and metastasis [147,148,149,150]. Genistein inhibits angiogenesis in vivo by downregulating the expression of VEGF and basic fibroblast growth factor (bFGF), the two crucial players in angiogenesis in RCC [151,152]. Sasamura et al., reported that genistein displays anti-proliferative action on RCC cell lines by inducing apoptosis [152]. Whereas, Majid et al., revealed that anti-proliferative action of genistein is due to cell cycle arrest at G2/M phase but not due to apoptosis [153]. They have reported for the first time that a tumor suppressor gene, *BTG3* (B-cell translocation gene 3) is epigenetically silenced in RCC and genistein can reactivate it by promoter demethylation and active histone modification [153]. miR-1260b, which is an oncogenic miRNA, is overexpressed in RCC which promotes cell proliferation and invasion, and inhibits several tumor suppressor genes associated with Wnt-signaling-induced tumorigenesis, such as *sFRP1*, *Dkk2* and *Smad4* [154]. Genistein downregulates miR-1260 expression and in turn, inhibits cell proliferation and invasion [154]. These studies support that genistein can be considered a promising agent for the treatment of RCC.

Sulforaphane (SFN) is an isothiocyanate derived from cruciferous vegetables such as broccoli (*Brassica oleracea*). SFN has been shown to play a bidirectional role; it acts as a protectant in normal kidney tubular cells against nephrotoxicants secreted by these cells, whereas it exhibits a pro-apoptotic effect on cancer cells by stimulating mitochondrial metabolism [155]. Moreover, studies found that SFN delays the resistance caused by chronic use of everolimus monotherapy and increases the efficacy of everolimus in RCC cell lines [156,157]. Further studies are warranted to verify these results in animal models.

Amygdalin (d-mandelonitrile-β-d-glucoside-6-β-glucoside), a cyanogenic substance is found in apricots, peaches, apple, cherry, plums, and other rosaceous fruit seeds [158]. Although a number of studies reported its anti-cancer properties in various cancers such as triple negative breast cancer, non-small lung cancer, prostate cancer, cervical cancer and liver cancer [158,159,160,161], very limited studies have been carried out to uncover its mechanism of action in RCC. Recently, it has been reported that amygdalin inhibits the growth of RCC cells by blocking adhesion and migration via an integrin-dependent mechanism [162,163].

Thymoquinone (2-methyl-5-isopropyl-1,4-benzoquinone), a monoterpene, is a natural polyphenolic compound found abundantly in the seed oil of black cumin (*Nigella sativa* L.) seeds and known to have anti-cancer properties [164]. Thymoquinone has recently been reported for its role in inducing apoptosis through downregulation of c-FLIP and BCL-2 in renal carcinoma cells [165]. Kahweol, a diterpene molecule from coffee beans has been reported to enhance the sensitivity to sorafenib in renal cell carcinoma cells through downregulation of MCL-1 and c-FLIP expression [166].

Alpinumisoflavone is isolated from *Erythrina lysistemon* and little is known about its anti-cancer effect in RCC. Recently, Wang et al., uncovered the mechanism of its anti-cancer effect suggested that this natural compound suppresses the tumor growth and metastasis through modulating miR-101/RLIP76 signaling [167].

16-hydroxycleroda-3,13-dien-15,16-olide, a clerodane diterpene (CD) isolated from *Polyalthia longifolia* var. pendula leaves has shown has been shown to inhibit the proliferation of various human cancer cell lines [168]. However, the mechanism of action of CD against RCC remains unknown. A recent study elucidated the mechanism of action of CD against RCC and suggested that it inhibits the cell proliferation and induces mitochondrial-dependent apoptosis through AKT, mTOR, and MEK/ERK pathways in RCC cells [169].

A very recent report demonstrates that Korean red ginseng extract can enhance the anticancer effect of sorafenib through suppressing cyclic adenosine monophosphate response element-binding protein and c-Jun activation, induce p53 phosphorylation and in turn enhances the chemosensitivity of sorafenib in RCC [170].

## 3. Conclusions

There are currently many agents available for the treatment of RCC such as anti-angiogenesis drugs (TKIs and bevacizumab) and immunotherapy drugs (interleukin and interferon). Renal cell carcinoma is one of the deadliest cancers, and, despite these many therapeutic options, is not curable in advanced stages. There is a clear necessity for medicines that are effective against the tumor while sparing the patient of adverse drug reactions. As an alternative approach, nature products have been proposed, but, to date, few of these compounds have been implemented on a large-scale in the treatment of cancer patients. Recent studies have suggested that many natural compounds are quite effective in vitro, and in vivo cancer models and history has shown this class of agent has little to no adverse side effects. EGCG, Englerin A, curcumin, resveratrol, quercetin, and honokiol are a few of these natural compounds that have shown beneficial results in preclinical studies of RCC. We have summarized the anticancer mechanism of these compounds in Table 1. It is prudent to continue to explore natural products as anti-tumor agents, without severe side effects, either alone or in rationally designed combination.

## Figures and Tables

**Table 1:** Anticancer Indications of Natural Products in Renal Cell Carcinoma.

Natural Products	Sources	Possible Targets	References
EGCG	Green tea, plums, apple peel, onions	TFPI-2, TRAIL, c-FLIP, MCL-1, BCL-2, MMP-2/9, *Cx32*, Src, JNK signaling	[28,36,37,43]
Englerin A	*Phyllanthus engleri*	AKT/ERK signaling, pathway, PKCθ, HSF1, TRPC4/5, E-cadherins, Vimentin, CD44, ALDH1A1	[44,47,48,49,50,51,52,56]
Quercetin	Tea, onions, grapes, and apples	miRNA-27a, COMT, ZBTB10, Snail	[65,66]
Coumarin	Strawberry, sweet grass, Tonka beans, Lavender	Caspase-9, G2/M phase	[82,83,84,86]
Curcumin	Rhizomes of turmeric plant	BCL-2, BCL-XL, IAP, caspase 3, cytochrome c, PARP, DR5, PI3K/AKT and mTOR signaling pathways	[91,113,114,115,116,122,123,125,126]
Resveratrol	Grapes, red wines	VEGF, STAT3/5, BCL-2, BCL-XL, IAP1/2, survivin, COX-2, MMP2, TGF-β, IFN-γ, IL-6 and IL-10	[133,135,136,137,138]
Honokiol	*Magnolia spp.* bark	RhoA/ROCK/MLC signaling pathways, miR-141, ZEB2, KISS-1, TIMP4, KISS-1R, TP53, CXCL12, CCL7, IL-18, and MMP7	[145,146]
Genistein	Soybeans	VEGF, bFGF, *BTG3*, miR-1260b, *sFRP1*, *Dkk2* and *Smad4*	[151,152,153,154]
Sulforaphane	Broccoli	Nrf2, PGC1α, HIF1α	[155]
Amygdalin	apricots, peaches, apple, cherry, plums	integrin α and β, FAK	[162,163]
Thymoquinone	Black cumin	c-FLIP and Bcl-2	[165]
Kahweol	Coffee beans	Mcl-1 and c-FLIP	[166]
Alpinumisoflavone	*Erythrina lysistemon*	miR-101/RLIP76 signaling	[167]
Clerodane diterpene	*Polyalthia longifolia*	Akt, mTOR, and MEK/ERK	[169]

EGCG, Epigallocatechin-3-gallate; TFPI-2, tissue factor pathway inhibitor-2; TRAIL, tumor necrosis factor-related apoptosis-inducing ligand; c-FLIP, FLICE-like inhibitory protein; McL-1, myeloid cell leukemia 1; Bcl-2, B-cell lymphoma 2; MMP, matrix metalloproteinas; *Cx32*, connexin 32; JNK, Jun N-terminal protein kinase; ERK, extracellular signal-regulated kinase; PKCθ, protein kinase C theta; HSF1, heat sock factor 1; TRPC, transient receptor potential cation channel; ALDH1A1, Aldehyde dehydrogenase 1 family, member A1; COMT, catechol-*O*-methyltransferase; ZBTB10, zinc finger and BTB domain-containing protein 10; IAP, inhibitor of apoptosis protein; PARP, poly ADP-ribose polymerase; DR5, death receptor 5; PI3K, phosphoinositide 2-kinase; mTOR, Mechanistic target of rapamycin; VEGF, vascular endothelial growth factor, STAT3/5, Signal transducer and activator of transcription 3/5; COX-2, cyclooxygenase-2; TGF-β, transforming growth factor β; IFN-γ, interferon gamma; IL, interleukin; ROCK, Rho-associated coiled-coil kinase; MLC, myosin light chain; KISS1, Kisspeptin; TIMP4, tissue inhibitors of metalloproteinase 4; CXCL12, Chemokine (C-X-C motif) ligand 12; CCL7, Chemokine ligand 7; bFGF, basic fibroblast growth factor; *BTG3*, B-cell translocation gene 3; *sFRP1*, Secreted frizzled-related protein 1; *Dkk2*, Dickkopf WNT Signaling Pathway Inhibitor 2; Smad4, Smad Family Member 4; Nrf2, Nuclear factor E2-related factor 2; PGC1α, proliferator-activated receptor-γ co-activator-1α; HIF1α, hypoxia-inducible factor-1α; FAK, focal adhesion kinase; c-FLIP, cellular FLICE (FADD-like IL-1β-converting enzyme)-inhibitory protein; RLIP76, Ral-interacting protein of 76 kDa.
